# A new approach to prediction riboflavin absorbance using imprinted polymer and ensemble machine learning algorithms

**DOI:** 10.1016/j.heliyon.2023.e17953

**Published:** 2023-07-12

**Authors:** Bita Yarahmadi, Seyed Majid Hashemianzadeh, Seyed Mohammad-Reza Milani Hosseini

**Affiliations:** a Real Samples Analysis Laboratory, Department of Chemistry, Iran University of Science and Technology, Tehran, Iran; b Molecular Simulation Research Laboratory, Department of Chemistry, Iran University of Science and Technology, Tehran, Iran

**Keywords:** Riboflavin, Machine learning, Ensemble algorithm, Molecularly imprinted polymer

## Abstract

The molecularly imprinted polymer (MIP) is useful for measuring the amount of riboflavin (vitamin B2), in various samples using UV/Vis instruments. The practical optimization of the MIP synthesis conditions has a number of drawbacks, like the need to spend money, the need to spend time, the use of the compounds that cause contamination, needing laboratory equipment and tools. Using machine learning (ML) to predict the amount of riboflavin absorbance is a creative solution to overcome the problems and shortcomings of optimizing polymer synthesis conditions. In fact, by using the model without needing real work in the laboratory, the optimum laboratory conditions are determined, and as a result the maximized absorption of the riboflavin is obtained. In this paper, MIP was synthesized for selective extraction of the riboflavin, and UV/Vis spectrophotometry was used to quantitatively measure riboflavin absorbance. Various factors affect the performance of the polymer. The effect of six important factors, including the molar ratio of the template, the molar ratio of monomer, the molar ratio of cross-linker, loading time, stirring rate, and pH, were investigated. Then, using ensemble ML algorithms, like gradient boosting (GB), extra trees (ET), random forest (RF), and Ada boost (Ada) algorithms, an accurate model was created to predict the riboflavin absorption. Also, the mutual information feature selection method was used to determine the important features. The results of using feature selection method was shown that variables such as the molar ratio of the template, the molar ratio of the monomer, and the molar ratio of the cross-linker had a high effect on riboflavin absorbance. The GB and Ada boost algorithms performed better than ET and RF algorithms. After tuning the n-estimator hyper parameter (n-estimator = 300), the GB algorithm was shown an excellent performance in predicting the absorbance of riboflavin and the maximum R^2^-scoring of the model was obtained at 0.965995, the minimum of the mean absolute error (MAE), and mean square error (MSE) of the model respectively were obtained −0.003711 and −0.000078. Therefore, by using the proposed model, it is possible to predict riboflavin absorbance theoretically, and with high accuracy by changing the inputs of model, and using the model instead of working in the lab saves time, money, chemical compounds, and lab ware.

## 1 Introduction

**Fig. 1:**
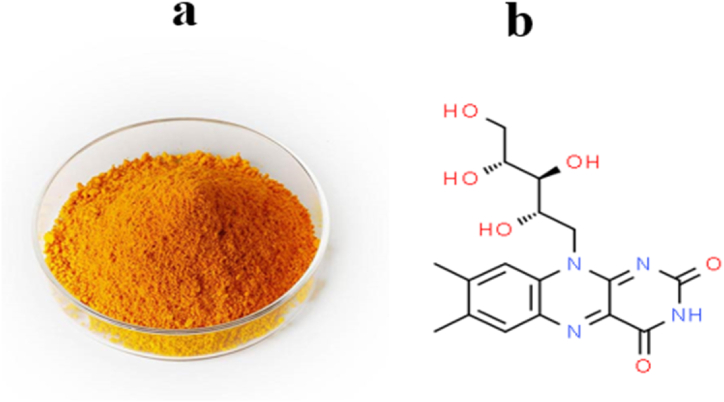
The physical properties (a) and chemical structure (b) of the riboflavin.

Riboflavin is an essential vitamin for human health. It is essential for breaking down food components, absorbing nutrients and maintaining tissues, and is found in grains, plants and dairy products. The function of this vitamin is antioxidant protection of the body against oxidizing substances [1]. Since the body can only store small amounts of this vitamin and the amount decreases quickly, people should take it every day. A deficiency of this vitamin caused by improper diet is a serious risk for a person because the human body constantly excretes this vitamin and does not store it [2]. The physical (a) and chemical structure (b) of riboflavin was shown in Fig. 1. Quantitative detection of riboflavin was done by different methods such as HPLC [3], fluorescence [4], UV/Visible spectroscopy [5], differential pulse voltammetry (DPV) [6], GC [7], and inductively coupled plasma atomic emission spectroscopy (ICP-AES) [8].

Solid phase extraction (SPE) has been developed and is used in various analytical fields, such as determining the amount of metals ions in water [9], malachite green in water samples [10], sildenafil citrate in water and biological samples [11], quercetin compound in fruit juice [12], aromatic compounds in different samples [13], chlorogenic acid in plants and water [14], lapis lazuli-II in aquatic environments [15] and mefenamic acid in biological samples [16]. The use of MIPs as SPE adsorbents is an exciting idea that improves some of the disadvantages of SPE adsorbents, such as poor selectivity [17]. The MIPs have particular recognition sites that resemble sorbents and specifically bind to a template molecule or other closely related compounds. MIP has advantages, such as low production cost, sensitivity, easy preparation, stability, and robustness [18].

In the last decade, ML has attracted the attention of many researchers, and has been used in various fields such as polymers [19], pharmaceuticals [20], medicine [21], drug delivery [22], sensors [23], etc. The use of ML has many advantages, including increasing speed, increasing accuracy, reducing time, and reducing cost [24].

Ensemble algorithms predict output with high accuracy, and these algorithms have attracted the attention of researchers in the field of polymer development. For instance, Mahmmad Nasir Amin et al. (2022), using this algorithm, created a very accurate model for geopolymers concrete mixtures with nine input variables, including sample age, curing time, Na_2_SiO_3_/NaOH ratio, temperature, alkali to fly ash ratio, NaOH molarity, super lubricant, aggregate volume, and water [25]. Also, Qichen Wang and colleagues (2022) used an individual ML approach, ensemble algorithms, including random forest, Adaboost, and decision tree to predict the compressive strength of polymer composites. According to their results in predicting the compressive strength of polymer composites, ensemble techniques perform better than individual techniques [26]. Devanshi Ledwani et al. (2022) create the models for predicting the melt flow rate of C3 and C2 polymers using Ada boost regressor, linear regression, and support vector regression. Their results showed that Ada boost regressor algorithm fittest with the maximum accuracy [27].

The GB Algorithm is another precise algorithm that uses multiple weak algorithms to create a more accurate algorithm. Therefore, by using it, instead of using an inadequate estimate or prediction, several strong algorithms are designed [28]. In recent years, the use of the GB algorithm has received much attention from researchers due to its advantages, such as high accuracy and speed in the development field and prediction of various properties of polymers. For example, Abdoulaye Sanni Bakouregui (2021) and co-workers presented a new approach to predict the bearing capacity of reinforced concrete (RC) columns reinforced with fiber-reinforced polymer (FRP) rods with an XGBoost algorithm. Their results showed that the proposed model works very well and is suitable for predicting the bearing capacity of FRP-RC columns [29]. Yuji Ueki et al. (2021) predicted the grafting efficiency for the radiation-induced graft polymerization of a methacrylate ester monomer to obtain a polyethylene-coated polypropylene fabric a target variable using several different algorithms. According to their results, among various algorithms as a prediction model in link performance, the GB model showed higher prediction accuracy compared to the multiple linear regression model [30]. Magzoub et al. (2021) used ML to develop a framework to identify the composition of materials for application in loss circulation, based on the desired rheological properties. They used four different algorithms for rheological modeling, including (a) k-nearest neighbor, (b) random forest, (c) GB, and (d) Adaboost. Their results showed that the XGBoost model had the highest accuracy and could be used for hydraulic calculations [31]. Hoang-Anh Le (2022) et al. developed a ML model using a GB algorithm to predict the shear strength (SS) of fiber-reinforced polymer (FRP). According to their results, this algorithm had a good accuracy for predicting shear strength [32].

The RF algorithm has also been used to predict and classify the properties of polymers. For example, Fredrik Skärberg et al. (2021) developed a segmentation method for data from porous ethyl cellulose films made from ethyl cellulose and hydroxypropyl cellulose (EC/HPC) polymer blends. They segmented the data using a random forest classifier. Their results showed good agreement with manual segmentations [33]. Kaffayatullah Khan and colleagues (2022) predicted the flexural capacity of flexural members with excellent accuracy using the RF regression model [34].

In addition, the use of the ET algorithm to predict the properties of polymers has been successfully reported. For instance, Gulnur Onsal et al. (2023) predicted the dielectric properties of the nematic liquid crystal (NLC) composite structures doped with phthalocyanine (*Pc*), using the ET algorithm. According to their results, this algorithm had the best prediction performance of dielectric constant values [35].

The main goal of this study is to develop a model that can predict the absorbance of the riboflavin with high accuracy based on the variables that affect the quality and stability of the MIP produced. The model created can be used to solve the shortcomings, and problems arising from the practical optimization of the MIPz synthesis conditions. The MIP was synthesized and used to quantitatively measure riboflavin with a UV/Vis spectrometer. After determining the absorbance of riboflavin under various synthesis conditions and preparing a dataset, an accurate model was developed to predict the absorbance of riboflavin using ensemble algorithms such as the ET, RF, GB, and Ada boost algorithms.

## 2 The materials and methods

### 2.1 The materials

All chemical compounds were of analytical grade, and all solutions were prepared with double distilled water (DDW). Tetraethyl orthosilicate (TEOS, 99.99%) and acrylamide (AA, ≥99.00%), 2, 2′-azobis (2-methyl propionitrile) (AIBN, 99.00%) were purchased from Merck (Darmstadt, Germany). Ethylene glycol methacrylate (EGDMA, 99.00%), hexadecyltrimethylammonium bromide (CTAB, ≥99.00%), ethanol, and hydrochloric acid were purchased from Sigma-Aldrich (www.sigmaaldrich.com, St. Louis, MO, USA). The pure riboflavin powder was obtained from Daru-pakhsh Company (www.dppharma.ir, Tehran, Iran).

### 2.2 The apparatus

A Biobase UV/Visible spectrophotometer model BK-UV1200, with 1-cm matched quartz cell was used to measure the absorbance of the riboflavin. A magnetic lab stirrer Model MR 2009 from Heidolph brand was applied in the polymer synthesis step. A surface evaluations were performed by a Zeiss scanning electron microscope (SEM) model S360.

### 2.3 The synthesis of MIP

The sol-gel polymerization method is a cost-effective method and the low reaction temperature allows good control over the chemical composition of the product. So this method was used for MIP synthesis as its steps are described below:

First, 0.8 ml DDW, 2 ml ethanol, 2 ml of TEOS, and 2 ml of hydrochloric acid (0.2 mol L-1) were mixed and stirred for 15 min. Then, 0.2 mg of acrylamide (functional monomer), 1.2 mg of AIBN (initiator), and 105 mg of EGDMA (cross-linker) were added, and the mixture stirring for 3 h at room temperature. Subsequently, the riboflavin molecules were dissolved in 1 ml of DDW, and the solution was stirred for 20 min at 34 °C. At the end of the procedure, 0.13 g CTAB and 4 ml ethanol were added to the mixture, and kept stirring for 4 h at ambient temperature. The synthesized MIP was used as a sorbent in further measurements. After preparing the polymer, a mixed solvent of water and acetonitrile in a ratio of 8:2 was used as a washing solvent. The spectrum of the samples was measured using a UV/Visible spectrometer [[36], [37], [38]].

### 2.4 The software and dataset

We used Python 3.9 software. Also, we used packages such as Scikit-Learn and SciPy, and libraries such as NumPy, matplotlib, and pandas. The dataset plays a vital role in modeling. In this study, dataset was obtained experimentally, that summarized in Table 1. The different factors, including pH, the molar ratio of the template, the molar ratio of the functional monomer, the molar ratio of the cross-linker, loading time, and string rate, were used as input. The riboflavin absorbance was used as output.

**Table 1:** The results of the synthesis conditions as dataset for modeling.

The molar ratio of the cross-linker	The molar ratio of the monomer	The molar ratio of the template	pH	Rate of stirring	Loading time	Absorbance
0.0032	0.0016	0.0002	6.2000	200.0	2.0000	0.0700
0.0037	0.0016	0.0002	5.3000	220.0	2.3000	0.0900
0.0043	0.0016	0.0002	6.2000	300.0	2.5000	0.0800
0.0048	0.0016	0.0002	5.4000	500.0	4.5000	0.1000
0.0054	0.0016	0.0002	5.0000	250.0	2.3000	0.1300
0.0059	0.0016	0.0002	5.2000	200.0	2.0000	0.0800
0.0064	0.0016	0.0002	6.0000	200.0	2.0000	0.0600
0.0048	0.0008	0.0002	7.000	200.0	2.0000	0.0700
0.0048	0.0010	0.0002	8.1000	250.0	2.4000	0.0890
0.0048	0.0013	0.0002	8.1000	300.0	2.5000	0.1400
0.0048	0.0016	0.0002	7.2000	500.0	4.0000	0.1000
0.0048	0.0018	0.0002	5.6000	200.0	2.6000	0.0600
0.0048	0.0021	0.0002	6.0000	200.0	2.1000	0.0201
0.0605	0.0201	0.0120	6.000	200.0	2.0000	0.0201
0.0512	0.0241	0.0120	7.2000	250.0	3.0000	0.0301
0.0832	0.0281	0.0120	5.0000	300.0	3.0000	0.0201
0.0763	0.0322	0.0120	5.200	500.0	2.700	0.0241
0.1521	0.0362	0.0120	6.0000	200.0	3.0000	0.0221
0.1033	0.0402	0.0120	6.2000	200.0	3.0000	0.0202
0.1002	0.0405	0.0120	7.2000	200.0	3.5000	0.0203
0.1001	0.0420	0.0120	5.7000	250.0	2.0000	0.0224
0.1022	0.0425	0.0120	8.1000	300.0	2.0000	0.0214
0.1022	0.0435	0.0120	6.2000	200.0	3.0000	0.0204
0.1000	0.0436	0.0120	7.3000	200.0	3.5000	0.0204
0.1000	0.0436	0.0120	7.4000	250.0	3.0000	0.0204
0.1000	0.0440	0.0130	7.3000	200.0	3.2000	0.0220
0.1000	0.0450	0.0170	7.2000	300.0	3.9000	0.0180
0.0048	0.0021	0.0002	6.0000	200.0	4.0000	0.0151
0.0048	0.0018	0.0002	5.6000	300.0	3.7000	0.0430
0.0048	0.0017	0.0001	5.6000	400.0	4.0000	0.0980
0.0048	0.0010	0.0027	5.8000	200.0	7.0000	0.0690
0.0048	0.0013	0.0027	6.7000	250.0	4.0000	0.1840
0.0048	0.0012	0.0027	5.8000	250.0	3.8000	0.0990
0.0048	0.0013	0.0027	7.0000	300.0	5.0000	0.0840
0.0048	0.0021	0.0002	4.6000	250.0	3.8000	0.0231
0.0048	0.0021	0.0027	4.6000	200.0	3.9000	0.0243
0.0048	0.0021	0.0027	4.6000	250.0	4.5000	0.0410
0.0048	0.0021	0.0027	4.6000	300.0	7.1000	0.0110
0.0048	0.0021	0.0027	4.6000	400.0	8.0000	0.0310
0.0048	0.0021	0.0027	4.6000	500.0	4.0000	0.0201

### 2.5 The modeling

In modeling, the feature selection step plays a vital role in increasing model accuracy and eliminating less valuable features. In general, reducing the number of the input variables leads to an increase in the model accuracy and a reduction in computational costs. The mutual information feature selection methods evaluate the relationship between input variables, and output variables, and examine the input variables that have the most robust relationship with the output variable. The next important step after determining the feature selection method is the determination of an appropriate and efficient algorithm. The ensemble algorithms have been efficient in the development of the polymers. The ensemble algorithms is one of the most powerful algorithms that can be used to predict not only a continuous target variable but also an absolute target variable. To create an accurate model for predicting the absorbance of unknown samples, four algorithms, including the GB, RF, ET, and Ada boost algorithms were used. Also, to divide the dataset into train and test data, the train-test-split function was used, and the ratio of train data to test data was 70.00 to 30.00. Pipelines were also used to improve model accuracy and prevent leakage of the training data to the test data, and vice versa. After determining the performance of the algorithms in predicting riboflavin absorption, the hyper parameters of the algorithm with the best performance were tuned. The inputs of the model were included the molar ratio of cross-linker, the molar ratio of functional monomer, the molar ratio of template, pH, rate of stirring, and loading time, and the output was riboflavin absorbance.

## 3 The result and discussion

### 3.1 The surface evaluation

**Fig. 2:**
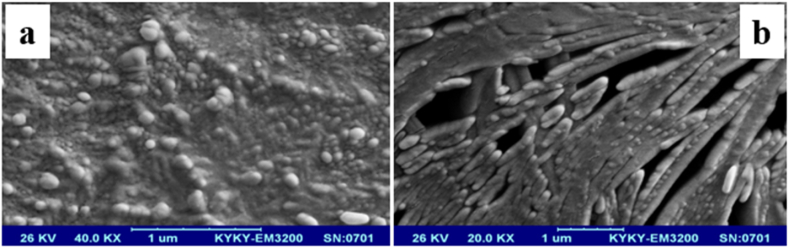
The SEM image of the MIP surface before (a) and after (b) washing with solvent.

A scanning electron microscope (SEM) analysis was used to evaluate the surface. The SEM image of the surface of MIP before (a) and after (b) washing with appropriate solvent was shown in Fig. 2. The SEM images show that after washing the MIP, holes corresponding to the size, and shape of riboflavin molecules remain. The optimum synthesis conditions, lead to excellent stability, and strong cavity retention, and MIP can be reused to extract riboflavin molecules.

### 3.2 The density plot

**Fig. 3:**
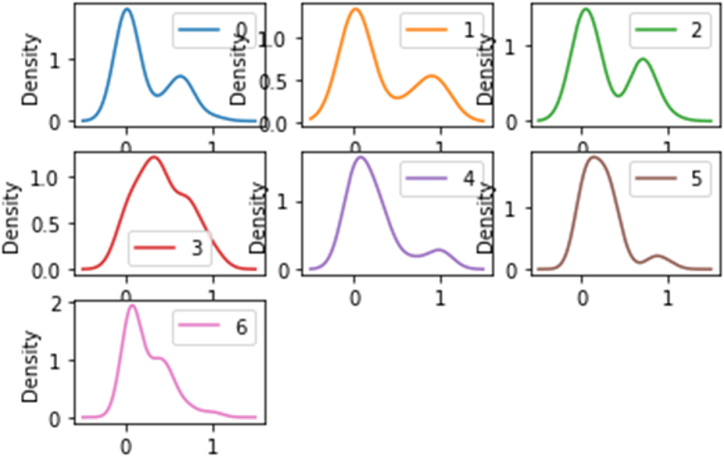
The density plots of the data from numbers 0 to 6, related to the molar ratio of the cross-linker, the molar ratio of the monomer, the molar ratio of the template, pH, stirring rate, loading time, and absorbance, respectively.

The distribution of data is one of the most critical factors that affect the accuracy of modeling. A density plot is a way to get a quick idea of the distribution of any feature. The density plot of the data is shown in Fig. 3. To the plot, the data have a wide distribution. The plots, from numbers 0 to 6, are related to the molar ratio of the cross-linker, the molar ratio of the monomer, the molar ratio of the template, pH, stirring rate, loading time, and absorbance respectively. The plot number 6, shows the distribution of the riboflavin absorbance with suitable range that means different amounts of the absorbance have been used to train the model, so the proposed model can predicts various amounts of the absorbance with high accuracy.

### 3.3 The Pearson correlation

**Fig. 4:**
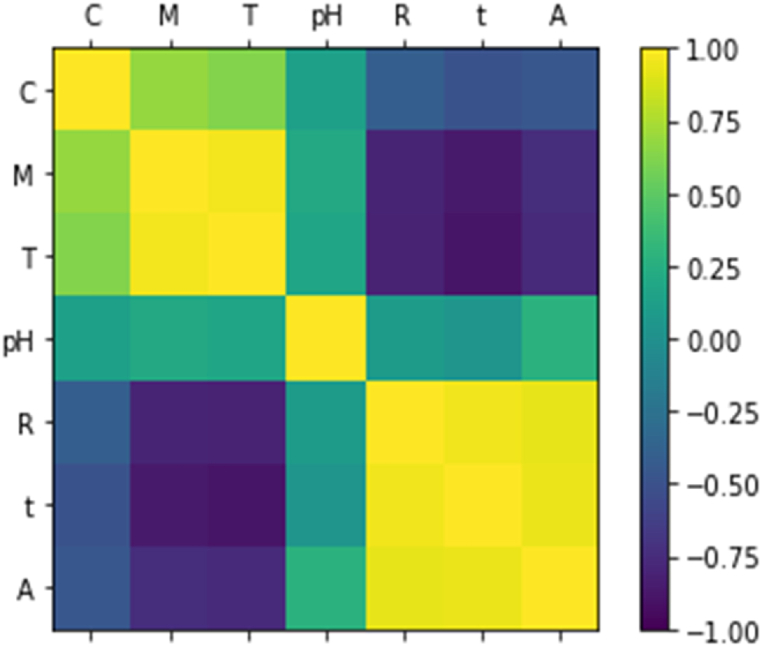
The Pearson correlation plot including C: the molar ratio of cross-linker, M: the molar ratio of functional monomer, T: the molar ratio of template, pH, R: rate of stirring, t: loading time, and A: absorption.

One of the important parameters in modeling is to check the correlation between variables. If the output and input of the model are highly correlated, it means that by input changing, the output value will also change, and depending on the sign (positive or negative), the change will be in the same direction or in the opposite direction. The Pearson correlation plot is shown in Fig. 4. According to the plot, yellow and purple colors indicate a high correlation between the two features. The output (absorption), has the highest correlation with time, and stirring rate (number one or yellow color). Also, the output has a high inverse correlation with the molar ratio of the template and the molar ratio of the functional monomer (negative number of one or purple color). The molar ratio of the cross-linker affects the riboflavin absorption, and pH has the minimum effect on output. The results of the correlation between different features are shown in Table 2 that confirmed the correlation plot. To the table, the correlation between different variables is represented by a number in the range of −1 to 1. The correlation increases as the number increases, and its sign indicates the direction of change of the two variables relative to each other. Time, stirring rate, the molar ratio of the template, the molar ratio of the functional monomer had a significant effect on the absorbance. The molar ratio of the cross-linker and pH had little effect on the riboflavin absorbance. The reason of the low correlation between the molar ratio of the cross-linker and absorbance is that in the sol-gel process, the sol gradually evolves towards the formation of a gel-like network that includes a solid phase and a liquid phase, and the metal alkoxide precursor (TEOS) undergoes hydrolysis and condense reactions. After adding cross-linker (EGDMA) and functional monomer (acrylamide), a network is formed which interacts with riboflavin from the side of the monomer molecules. Therefore, the molar ratio of the cross-linker has less effect on the absorbance than the molar ratio of the template and functional monomer. Since the synthesis pH changes were in a limited range (4.600–8.100), we expected that the pH changes would not significantly affect the imprinting quality, also would have little correlation with the model output, and the results confirmed this issue.

**Table 2:** The correlation results between different features.

Feature	The molar ratio of the cross-linker	The molar ratio of the monomer	The molar ratio of the template	pH	Stirring rate	Loading time	Absorption
The molar ratio of the cross-linker	1.000000	0.681383	0.632671	0.132297	−0.403822	−0.497176	−0.454332
The molar ratio of the monomer	0.681383	1.000000	0.964922	0.208772	−0.791782	−0.852472	−0.735756
The molar ratio of the template	0.632671	0.964922	1.000000	0.176657	−0.801447	−0.884065	−0.763877
pH	0.132297	0.208772	0.176657	1.000000	0.095349	0.042667	0.279382
Stirring rate	−0.403822	−0.791782	−0.801447	0.095349	1.000000	0.953671	0.928234
Loading time	−0.497176	−0.852472	−0.884065	0.042667	0.953671	1.000000	0.943597
Absorption	−0.454332	−0.735756	−0.763877	0.279382	0.928234	0.943597	1.000000

### 3.4 The feature selection results

**Fig. 5:**
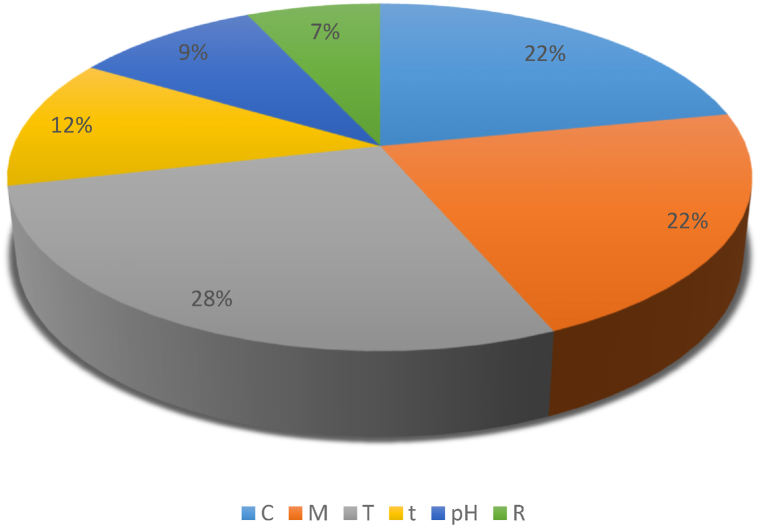
The results of the using mutual information feature selection method.

The mutual information feature selection method was used to determine the most important features affecting the absorption. The results of using this feature selection method are shown in Fig. 5. Using this method assigns a score to each feature. If a higher score is assigned to a feature, the feature has more effect on the absorption. According to the results, the molar ratio of the template (T), the molar ratio of the functional monomer (M), and the molar ratio of the cross-linker (C) had the maximum effect on the absorbance. After that, loading time (t), and pH were effective on the absorbance, and the stirring rate (R) had minimum effect on the absorption. Due to the pH of the solution changed in the range of 4.600–8.100, this feature had little effect on the output. According to Table 2, stirring rate and loading time were highly correlated with absorbance. These two features were assigned low scores in the feature selection results, because their small changes cause large changes in the absorbance, and they control the kinetics of the synthesis reaction. If the effect of these two features on the output is considered, their high correlation with output caused the effect of other features on the output is ignored.

### 3.5 Prediction using algorithms

**Fig. 6:**
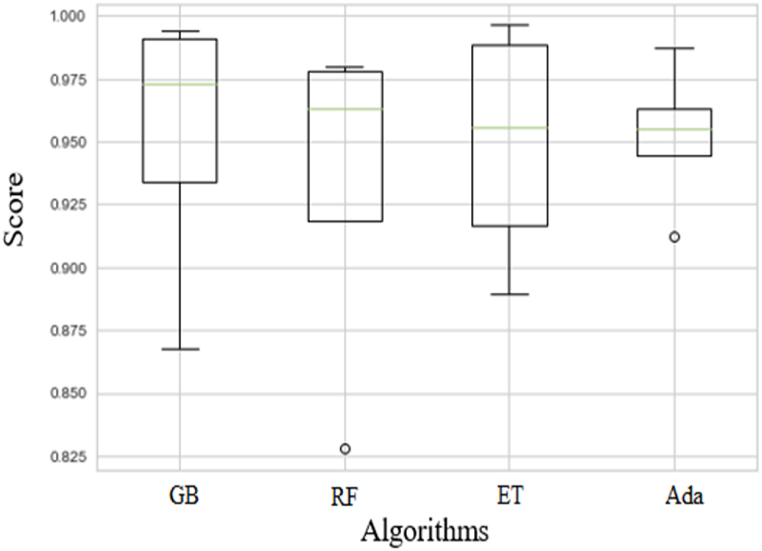
The box plot of the R2-scoring for the algorithms including GB, RF, ET, and Ada.

The results of using ensemble algorithms for modeling are shown in Table 3. In general, these algorithms showed a good performance in predicting the absorbance of the samples. In order to ensure the performance of the algorithm, the results were repeated five times and the average value was reported, and R2-scoring, MAE and MSE were used to evaluate the efficiency of the algorithm. The GB and Ada boost algorithms showed very good accuracy in predicting riboflavin absorption. The highest accuracy was related to the GB algorithm, and the R2-scoring was obtained 0.953911, and the average MAE and MSE were obtained −0.004117 and −0.000079, respectively. The lowest accuracy was related to the RF algorithm. The numbers written in parentheses show the standard deviation of the calculation. In addition, a box plot of the R2-scoring for visual comparison of the results when using different algorithms is shown in Fig. 6. In fact, this plot clearly shows the results of the first column of Table 3, and summarizes the distribution of the R2-scoring values. The green line inside the box represents the median of the data, and the points outside the lines represent the outliers. The use of the GB algorithm resulted in the highest average R2-scoring, and the absence of outliers.

**Table 3:** The results of using the ensemble algorithms for modeling.

Algorithm	R^2^	MAE	MSE
GB	0.953911 (0.050831)	−0.004117 (0.002840)	−0.000079 (0.000122)
RF	0.933382 (0.062133)	−0.005891 (0.002752)	−0.000161 (0.000190)
ET	0.949196 (0.043693)	−0.003221 (0.002363)	−0.000088 (0.000111)
Ada	0.951290 (0.026525)	−0.005347 (0.002196)	−0.000083 (0.000078)

### 3.6 The GB algorithm tuning

The n-estimators is a hyper parameter for increasing GB algorithm performance. By changing the value of the n-estimators, the value of R2, MAE, and MSE also changes. A value of the n-estimators that results in the highest values of R2, and the lowest values of MAE, and MSE is appropriate. The results of tuning n-estimators hyper parameter are shown in Table 4. The default number of n-estimators is 100. Often a larger number of n-estimators leads to the better performance of the GB algorithm. To adjust the n-estimators hyper parameter, we defined its values from 50 to 600. To the results when the n-estimators was set to 300, the best performance was obtained, and the model had the maximum value of R^2^ (R^2^ = 0.965995), and the minimum value of the MSE (MSE = −0.000078), and MAE (MAE = −0.003711). The numbers written in parentheses show the standard deviation of calculate.

**Table 4:** The results of the tuning the n-estimator values.

n-estimators	R^2^	MAE	MSE
50	0.943955 (0.049808)	−0.003921 (0.002863)	−0.000161 (0.000160)
100	0.944125 (0.032333)	−0.004910 (0.003110)	−0.000145 (0.000340)
150	0.945770 (0.03220)	−0.004907 (0.003542)	−0.000151 (0.000110)
200	0.926654 (0.037801)	−0.004902 (0.003460)	−0.0001771 (0.000140)
250	0.948850 (0.029808)	−0.003900 (0.0036601)	−0.000080 (0.000042)
300	0.965995 (0.040008)	−0.003711 (0.002002)	−0.000078 (0.000085)
350	0.953115 (0.032105)	−0.004115 (0.003811)	−0.000079 (0.000064)
400	0.951110 (0.022118)	−0.004201 (0.003017)	−0.000081 (0.000055)
45O	0.940055 (0.036607)	−0.005177 (0.003114)	−0.000082 (0.000076)
500	0.930066 (0.051200)	−0.005262 (0.002772)	−0.000087 (0.000990)
550	0.936177 (0.043201)	−0.005212 (0.002441)	−0.000096 (0.000997)
600	0.931805 (0.049003)	−0.005267 (0.002532)	−0.000088 (0.000083)

### 3.7 Prediction error plots

**Fig. 7:**
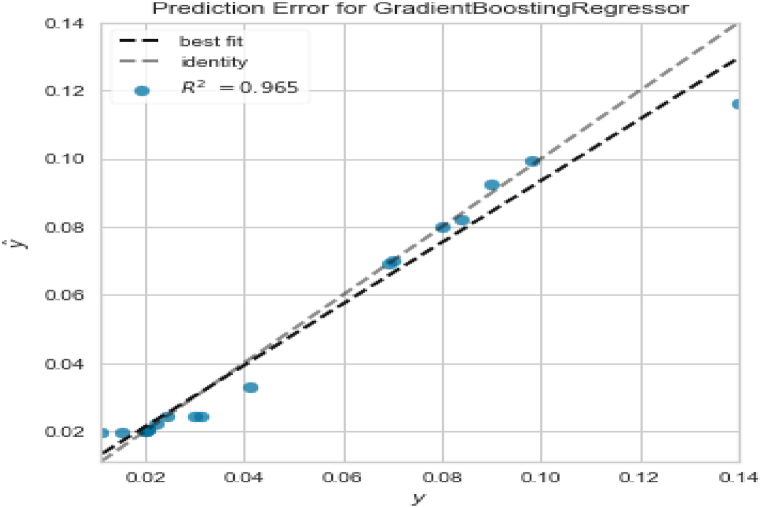
The prediction error plot of the model after tuning n-estimator hyper parameter (n-estimators = 300).

A prediction error plot for the model is shown in Fig. 7. The x-axis, show the actual values and the y-axis show the predicted values for each absorption. If the best fit line and the identity line are coincident, the modeling accuracy will be excellent. Factors like hyper parameter settings, pipeline usage, feature selection method, and the type of the algorithm affect modeling accuracy. This plot was obtained after adjusting these factors. A pipeline was used to prevent training data from entering the test data, and vice versa. The mutual information feature selection method and a GB algorithm (n-estimators = 300) were also used. To the plot, the highest modeling accuracy was obtained (R^2^ = 0.965).

## 4 Conclusion

Using ML and predicting riboflavin absorbance in different synthesis conditions is a suitable solution to overcome the shortcomings, and practical optimization problems of MIP synthesis conditions. Manually changing the model inputs can change the model output or absorption rate, so building a model that can accurately predict riboflavin absorption is of great value. The MIP for riboflavin detection by UV/Visible spectroscopy was synthesized by sol-gel polymerization under optimal synthesis conditions. The effect of six important factors including the molar ratio of template, the molar ratio of monomer, the molar ratio of cross-link, loading time, stirring rate and pH were investigated. According to the results of using the mutual information feature selection method, the molar ratio of the template, the molar ratio of the monomer and the molar ratio of the cross-link had the greatest effect on the imprinting quality. In fact, the stability of a polymer depends on the degree of interaction and intermolecular forces between the template and functional monomer molecules. By changing these ratios, the optimal ratio that leads to quality printing was obtained. In this work, ensemble algorithms including GB, RF, ET, and Ada boost algorithms were used for modeling. A high-accuracy model was created using the GB algorithm to predict the amount of riboflavin absorption. After adjusting the n-estimators hyper parameter of the GB algorithm, the maximum R^2^-scoring of model was obtained 0.965995, and the minimum MAE and MSE of model respectively were obtained −0.003711 and −0.000078. The Ada boost algorithm also had a very good performance for predicting riboflavin absorbance and using this algorithm leads to obtain acceptable value of the R^2^-scoring (R^2^ = 0.951290). Also the R^2^-scoring values for ET and RF algorithms were obtained as 0.949196 and 0.933382, respectively. The use of ML in the development of the polymers is a new research issue that will lead to many advances in the development of future polymers. For further investigation in this field, it is suggested to consider the effect of other input variables like the ratio between washing solvents in the output, or to predict the output of several analytical devices (for example fluorescence and UV/Vis output) simultaneously through the model.

## Ethics approval and consent to participate

The authors certify that the following conditions are met for the manuscript:

1) The written content is the original work of the author and has not been published elsewhere.
2) Currently, this article has not been submitted for publication in another journal.
3) The content of this article is the result of the authors' own research and analysis
4) All authors have actively contributed to the work leading to the article and take general responsibility for its content.

## Consent for publication

The authors declare their consent to publish all the content in this article, including photos, designs and details within the text (materials, synthesis method and the result of using feature selection methods).

## Funding

There are no funds.

## Author contribution statement

Bita Yarahmadi: Conceived and designed the experiments; Performed the experiments; Analyzed and interpreted the data; Contributed reagents, materials, analysis tools or data; Wrote the paper.

Seyed Majid Hashemianzadeh: Conceived and designed the experiments; Analyzed and interpreted the data; Contributed reagents, materials, analysis tools or data; Wrote the paper.

Seyed Mohammad-Reza Milani Hosseini: Performed the experiments; Analyzed and interpreted the data; Contributed reagents, materials, analysis tools or data.

## Data availability statement

Data included in article/supplementary material/referenced in article.

## Additional information

No additional information is available for this paper.

## Declaration of competing interest

The authors declare that they have no known competing financial interests or personal relationships that could have appeared to influence the work reported in this paper.
